# Hybrid Zero Dynamics Control for Gait Guidance of a Novel Adjustable Pediatric Lower-Limb Exoskeleton

**DOI:** 10.3390/bioengineering9050208

**Published:** 2022-05-12

**Authors:** Anthony Goo, Curt A. Laubscher, Jason J. Wiebrecht, Ryan J. Farris, Jerzy T. Sawicki

**Affiliations:** 1 Department of Mechanical Engineering, Washkewicz College of Engineering, Cleveland State University, Cleveland, OH 44115, USA; a.goo@vikes.csuohio.edu (A.G.); c.laubscher@csuohio.edu (C.A.L.); j.wiebrecht@vikes.csuohio.edu (J.J.W.); 2 Department of Engineering, School of Science, Engineering, and Health, Messiah University, Mechanicsburg, PA 17055, USA; rfarris@messiah.edu

**Keywords:** pediatric exoskeleton, hybrid zero dynamics, robot control

## Abstract

Exoskeleton technology has undergone significant developments for the adult population but is still lacking for the pediatric population. This paper presents the design of a hip–knee exoskeleton for children 6 to 11 years old with gait abnormalities. The actuators are housed in an adjustable exoskeleton frame where the thigh part can adjust in length and the hip cradle can adjust in the medial-lateral and posterior-anterior directions concurrently. Proper control of exoskeletons to follow nominal healthy gait patterns in a time-invariant manner is important for ease of use and user acceptance. In this paper, a hybrid zero dynamics (HZD) controller was designed for gait guidance by defining the zero dynamics manifold to resemble healthy gait patterns. HZD control utilizes a time-invariant feedback controller to create dynamically stable gaits in robotic systems with hybrid models containing both discrete and continuous dynamics. The effectiveness of the controller on the novel pediatric exoskeleton was demonstrated via simulation. The presented preliminary results suggest that HZD control provides a viable method to control the pediatric exoskeleton for gait guidance.

## 1. Introduction

Gait abnormalities in the pediatric population can arise as a secondary effect of various disorders such as spina bifida, muscular dystrophy, and cerebral palsy. Therapeutic intervention to address walking impairment is critical to mitigate the progression of gait disability, especially for the pediatric population [1]. Traditional physical therapy approaches include treadmill and overground gait training, sometimes with walking aids or with an accompanying overhead harness system for partial alleviation of patient weight [1,2]. Therapists manually move the patient limbs to guide leg motion while the subject walks. Such approaches have seen clinical application for rehabilitation within the pediatric population, with improved outcomes observed in gross motor function, walking ability, and step length [2]. However, such approaches are limited in that they can be asymmetrical between the two legs of the patient [3], can have insufficient exertion to provide adequate rehabilitation [4], and can be physically exhausting for the therapist, thereby limiting therapy session length [5].

Lower-limb exoskeletons are powered orthotic devices capable of providing torque to the patient joints. Such devices have been suggested for providing robot-assisted gait training to alleviate the aforementioned limitations of traditional approaches. In addition, other benefits include improved repeatability in applied torque or motion profile and direct measurement of gait which can be used for automatic quantitative assessment. Many devices are currently available for the adult population, as presented in other review articles [5,6]. Relatively few exoskeletons have been developed for children and each with their own limitations [7,8]. Devices such as the Walkbot-K, the Giergiel et al. device, the CPWalker, and the pediatric version of the Lokomat are either stationary or used in conjunction with large mobility devices, thereby making them impossible or impractical for broader use [7]. The ideal exoskeleton should be applicable not only for therapeutic intervention in a clinical setting but also for assistance in a community environment. Non-stationary devices such as the one presented by Copilusi et al. [9] or Lerner et al. [10] only actuate a single degree of freedom (DoF), limiting their generalizability for addressing gait issues across multiple joints.

There are two devices that alleviate these limitations. The ATLAS 2020 exoskeleton device is a highly actuated system with five actuated DoF per leg: hip flexion-extension, hip abduction-adduction, knee flexion-extension, ankle dorsiflexion-plantarflexion, and ankle eversion-inversion [11]. The adjustable device can fit children 3 to 11 years old, but is bulky and heavy at around 12 kg due to the highly actuated nature of the exoskeleton. The exoskeleton’s weight represents a significant burden on younger children as the exoskeleton comprises a large proportion of the total patient-exoskeleton system weight. The P-LEGS exoskeleton is a comparatively lighter device at 8 kg, as it only actuates three sagittal DoF per leg. Additionally, the device targets a smaller age range from 4 to 8 years old [12]. The design of this exoskeleton consists of several motor modules that are linked together via 3D printed braces of varying lengths. While this allows for subject-specific customization of the device, the repetitive disassembly and reassembly of the device precludes it from use in a clinical setting where multiple children with varying anthropometrics may need to use the same device. There is presently a need for a pediatric exoskeleton that is adjustable, lightweight, and requires minimal reassembly for use between patients of differing anthropometrics.

Control of exoskeletons for rehabilitation has primarily focused on enforcing a trajectory along some reference gait [13]. The reference can be derived from motion capture data obtained by the researchers or reported in the literature for adults [14] or children [15]. This can be formulated in a time-invariant manner [16], which gives more volitional control to the user, thereby making the exoskeleton easier to use and more comfortable as compared to time-dependent references. However, the reference data derived from motion capture or similar methods have no theoretical guarantee of dynamic stability for a person wearing an exoskeleton. A dynamically stable gait is one in which the combined human-exoskeleton walking cycle is periodic yet may include portions of unbalanced motion such as momentary falling phases [17]. Such motions are observed in nominal human gait and are a desirable property in motion control in exoskeleton systems [18]. Hybrid zero dynamics (HZDs) can provide dynamically stable reference gaits. This approach uses feedback control to drive the system toward an optimally defined manifold and ultimately enforces virtual constraints on the system. The approach was originally applied in biped robots such as the RABBIT, DURUS, ERNIE, and MABEL devices [19,20]. More recently, HZD has also been applied to exoskeleton systems. This allows for increased volitional control by the subject due to time-invariant feedback control implementation while also following a dynamically stable gait profile. HZD has been applied in a fully actuated exoskeleton systems as seen in the research conducted with the Wandercraft Atalante [21,22]. In addition, the concepts of HZD have been extended to an underactuated exoskeleton system for providing gait guidance in our prior studies [23,24].

This paper presents significant contributions toward the development and control of a pediatric lower-limb exoskeleton for gait assistance and rehabilitation with the design of an adjustable pediatric exoskeleton frame that can be reconfigured to fit the anthropometrics of children between the ages 6–11 years old, and the application of a HZD-based controller to a pediatric human-exoskeleton system in simulation. The adjustable exoskeleton frame was designed to utilize the developed actuator discussed in the authors’ prior work [25]. This paper also extends the authors’ prior work on HZD control of exoskeletons for adults to the pediatric population and child-oriented devices [23,24]. The contents of this paper are organized as follows. Section 2 describes the target population and their implications on the exoskeleton design, reviews the joint actuator design and characteristics, and explains the design of the adjustable exoskeleton frame. Section 3 discusses the expected gait and spatiotemporal parameters. Section 4 discusses the generation of the hybrid model to be used in the HZD framework and pediatric simulation. Section 5 describes the HZD framework, including the application of virtual constraints and the optimal generation of a reference zero dynamics manifold. Section 6 presents the simulation results of the application of HZD to the modeled pediatric patient-exoskeleton system. Section 7 concludes with final remarks and future research areas of pursuit.

## 2. CSU Pediatric Exoskeleton Actuator

### 2.1. Target Patients and Anthropometrics

The target indication for the pediatric exoskeleton is children 6 to 11 years old with gait disorders such as cerebral palsy [26,27]. The authors chose to design around nominal anthropometric parameters available from the literature, with the most recently published census data shown in Table 1 [28]. The exoskeleton should be designed such that it is able to accommodate the majority of children within the weight range of 23.9 to 46.5 kg and height range of 118.8 to 149.3 cm.

For the purpose of gait analysis and simulation, the body was composed of five rigid body segments: one head–arms–torso (HAT), two thighs, and two shanks with the feet included. Normalized body segment parameters were taken from the literature [29] which were used with the gross anthropometrics from Table 1 to calculate the body parameters as shown in Table 2. An average 8 year-old child model was used in the later-described simulation and thus, Table 2 only tabulates anthropometrics for the single age.

### 2.2. Joint Actuator

**Figure 1:**
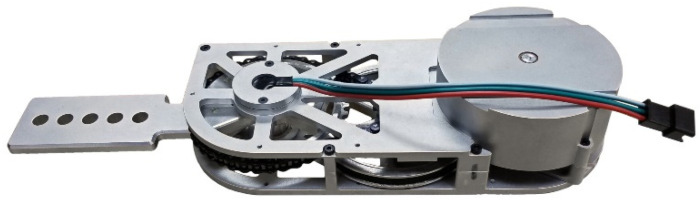
Photograph of the fabricated joint actuator for the pediatric exoskeleton.

The pediatric exoskeleton under development is powered at the hip and knee joints using identical modular actuators, shown in Figure 1. The design requirements, hardware description, and performance evaluations of the exoskeleton joint actuator were published in our prior study [25]. These actuators are in turn a design iteration upon a previous exoskeleton joint actuator [26,30,31]. In broad terms, the actuators had to be lightweight, modular to allow for ease of maintenance, and accommodate the expected range of motion and joint velocities typical of human gait. The actuator must also provide enough torque and power to significantly assist human walking motions. Each actuator weighs 0.45 kg and is powered by a 144 W brushless DC motor. This is transmitted through a belt and chain transmission to scale the torque up by a factor of 20.4:1. Experimental benchtop testing showed that the output of the actuator could achieve up to 1176 deg/s of rotational speed under no load, much greater than that in nominal gait [14,28]. The actuators were tested up to 21.1 Nm in peak value and were rated for 5.9 Nm of continuous torque, which constitutes a significant portion of the torques expected in nominal walking in a child [14,28]. The actuator evaluations also included a test to identify the effects of joint friction on the actuator output during operation, and the identification of a simple frictional model consisting of Coulomb, viscous, and static friction. In these evaluations, the kinetic friction effects yielded a frictional resistive torque of less than 0.5 Nm when the actuator was moving at the expected maximum operating velocity of 400 deg/s. Viscous friction levels were reported at 0.0188 Nm·s/rad with Coulomb friction levels around 0.212 Nm and static friction levels at 0.403 Nm at the actuator output. This roughly equates to a 62% and a 69% reduction in Viscous and Coulomb friction, respectively, with respect to the previous iteration of the actuators [25]. Compared to actuators for other orthotic devices, the friction values recorded represent a low passive resistance and friction levels for an exoskeleton joint actuator [32]. Additionally, the reflected inertia post-transmission of the actuator was experimentally identified in Ref. [25] at $1.45\times 10^{-3}$ kg·m^2^, which represents a 49% reduction in inertia with respect to the previous iteration of the actuators. Prior work with the previous iteration of the actuators in an anthropometrically parametrized exoskeleton demonstrated the backdrivability of the previous iteration of the actuators [31]. Thus, the low joint actuator frictions, the decrease in reflected inertia with respect to the previous actuators, and the ease at which the actuator output was able to be manipulated by hand indicates easy backdrivability of the actuators and by extension, the eventual exoskeletal system.

### 2.3. Adjustable Frame Components

**Figure 2:**
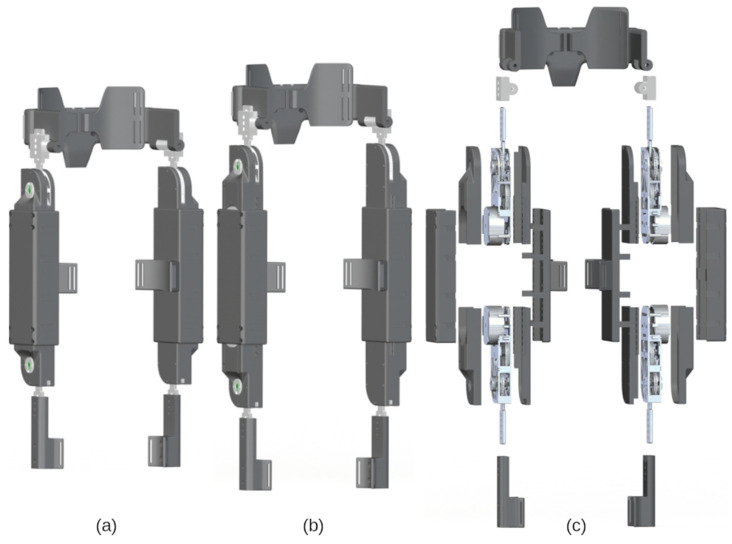
Renderings of the pediatric exoskeleton: assembled view for a child (**a**) 6 years old and (**b**) 11 years old, and (**c**) exploded view exhibiting the actuators and adjustable frame components.

**Figure 3:**
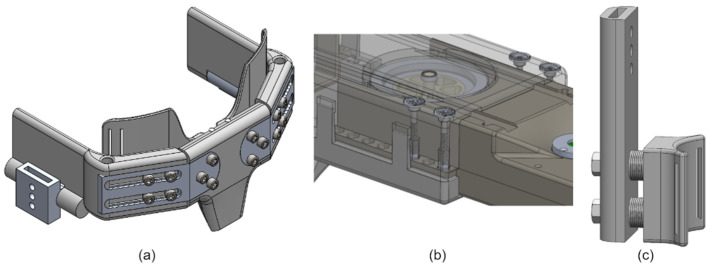
CAD model snapshots exhibiting the adjustability of the (**a**) hip cradle subassembly, (**b**) thigh subassembly, and (**c**) shank subassembly.

With the actuator design established, what remained was the design of a pediatric exoskeleton frame that is lightweight and is capable of link adjustment. The adjustability must allow for the exoskeleton to fit children 6 to 11 years old with minimal disassembly and reassembly. The exoskeleton design is discussed in terms of its three primary subassemblies: the hip cradle, the thigh, and the shank. The exoskeleton is portrayed in Figure 2, configured for children 6 and 11 years old and in an exploded view. The range of adjustment for each subassembly and the expected range of subject anthropometrics are listed in Table 3. Representative snapshots of each of these subassemblies are shown in Figure 3. With respect to materials, the metal components are primarily machined from aluminum 7075 whereas the plastic parts are 3D printed from an acrylate-based plastic. Table 4 includes a parameter list for each of the primary subassemblies when configured for an average 6, 8, and 11 year-old child.

#### 2.3.1. Hip Cradle Subassembly

The hip cradle consists of seven total parts: a backplate, two aluminum arms, two hip side plates, and two torso wings. The backplate is attached to two aluminum arms, upon which the two hip side arms slide to adjust hip cradle width and depth. Screws, threaded inserts, and geometry features on the aluminum bar allow the various components to lock into place. The torso wings are attached to the hip side arms by a rotating shaft, and help affix the hip cradle onto the subject’s torso using straps.

#### 2.3.2. Thigh Subassembly

The thigh subassembly houses the actuators developed in [25] and consists of separate shells for the actuators and a larger tube-like structure within which the actuators can slide. The top and bottom actuator shells screw directly onto the aluminum housing of the actuator. The bottom shell has tabs running along the sides with hex-shaped geometries cut out at 1 cm intervals. The actuator assemblies can then slide along the major axis of the thigh shell at both ends and can be locked in place via screw mechanisms that raise or lower hex nuts into the actuator shell hex hole geometries. A thigh cuff is included to fixate the thigh subassembly onto the subject body segment.

#### 2.3.3. Shank Subassembly

The shank subassembly of the exoskeleton does not need to adjust along the proximal-distal axis of the link. The shank subassembly only needs to translate forces provided by the knee actuator torques onto the shank of the subject. To facilitate this, a shank extension arm is affixed to the actuator arm. Along the shank extension arms are screw holes which allow modular cuffs to be added. These cuffs can project off of the shank arm and allows the components to fix and translate torque from the knee actuators onto the lower leg of the subject via straps.

## 3. Reference Gait and Spatiotemporal Parameters

**Figure 4:**
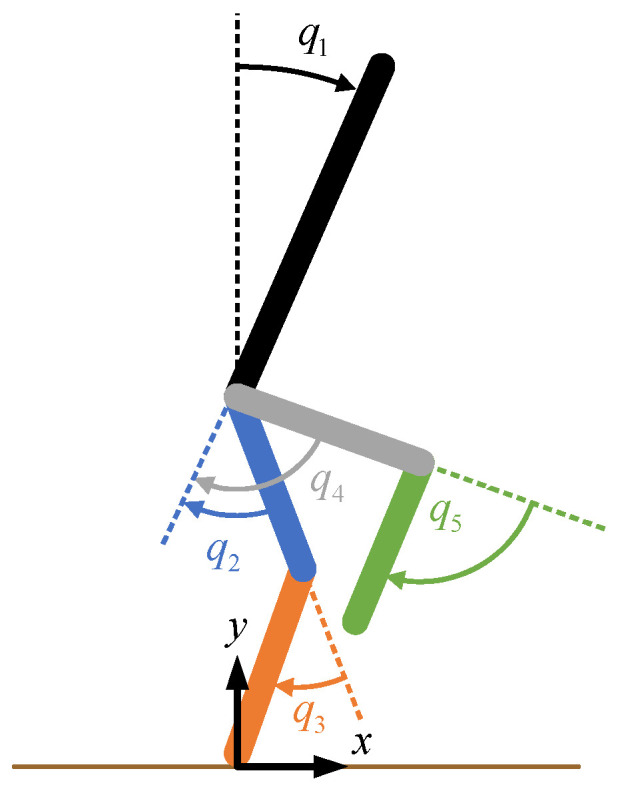
Joint diagram demonstrating the measurement conventions. For the hips, positive values correspond to hip extension. For the knees, positive values correspond to knee flexion.

**Figure 5:**
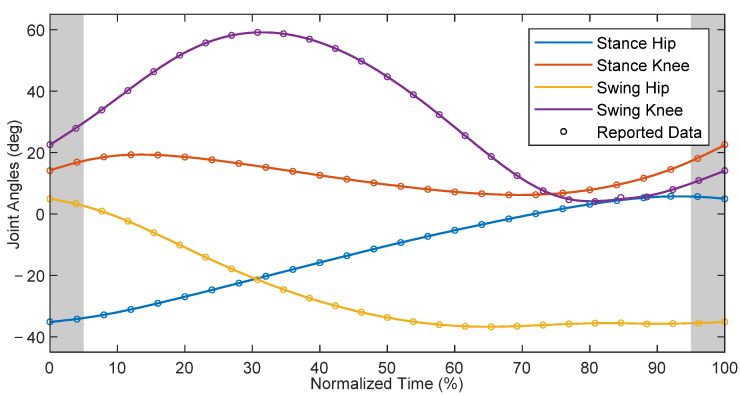
Time-normalized reference gait for children, where the shaded region represents the average double-supported portions of the gait.

For the purpose of trajectory tracking via HZD control, nominal data for healthy children were chosen as a reference [15]. These data were adjusted to match the measurement convention shown in Figure 4, which was also used in the model defined in Section 4.2. To summarize, clockwise rotations of the links in the orientation shown relative to the proximal joints represent positive rotations. To make the reference data compatible with the later-discussed hybrid zero dynamics control simulation, the gait data presented in Figure 5 start shortly before swing foot toe-off and end shortly after swing foot heel-strike. The trajectory corresponds to the gait patterns of pediatric subjects walking at a comfortable self-selected speed of approximately 1.0–1.4 m/s [15]. These patterns represent the target trajectory for a subject assisted by the exoskeletal system.

The spatiotemporal gait parameters from the literature are provided as values normalized to be dimensionless quantities [15]. The step length *S*, cadence *C*, and speed *v* are related to their dimensionless counterparts, respectively denoted as $S^{*}$, $C^{*}$ and $v^{*}$, through

(1) $$S=S^{*}L$$

(2) $$C=C^{*}\sqrt{g/L\;}$$

(3) $$v=v^{*}\sqrt{gL}$$

where *g* is the acceleration due to gravity and *L* is the hip height of the subject. Knowing the standing height of the target patients from Table 1 and scaling to get the hip height through a standard body proportion [29], the denormalized values could be calculated. The step length and speed are shown in Table 5.

The cadence of children is typically greater than that of adults, upwards of 144 steps per minute [14]. Cadence (in steps per second) is used to calculate the single support gait period *P* using

(4) $$P=\frac{1}{C},\;\;\;\;\;\;\;\delta P=\left|\frac{\partial P}{\partial C}\right|\delta C=\frac{1}{C^{2}}\delta C$$

where *δC* is the recorded standard deviation of the cadence and *δP* is the calculated standard deviation of the period, which are also reported in Table 5. The speed, step length, and step period can be used as guiding factors for the constraints in the phase-dependent reference optimization described in Section 5.

## 4. Patient-Exoskeleton Hybrid Model

For control and simulation, it is necessary to have a model of the entire patient-exoskeleton system. A planar robot model was derived based on rigid body dynamics. The parameters of the links in the connected rigid bodies are found in Section 4.1, which were subsequently used in deriving the equations of motion using Lagrangian mechanics in Section 4.2.

### 4.1. Model Parameters

The human body segment parameters were previously identified in Table 2, and the exoskeleton component parameters were previously identified in Table 4. The body segment parameters of the subject can be combined with the corresponding component parameters of the exoskeleton to create a rigid body model of the combined human-exoskeleton system. The parameters of the rigid links comprising this model could be calculated through the following equations.

(5) $$m=m_{1}+m_{2}$$

(6) $$x=\frac{m_{1}x_{1}+m_{2}x_{2}}{m_{1}+m_{2}}$$

(7) $$I=I_{1}+I_{2}+\frac{m_{1}m_{2}}{m_{1}+m_{2}}{\left(x_{1}-x_{2}\right)}^{2}$$

For each link of the human-exoskeleton system, the mass of the link is represented by the scalar m, while the mass of the two constituent components (human and exoskeleton parameters) are denoted as scalars $m_{i}$. It is assumed that the CoM location lies along the primary axis of each link. Thus, the location of each link’s CoM relative to the proximal joint along the proximal-distal length of the link is represented by the scalar value x, with the CoM locations of the constituent components denoted as the scalars $x_{i}$. Similarly, the moment of inertia of the combined link about its CoM is represented as the scalar I, and is calculated as the sum of the inertias of the constituent components, $I_{i}$ about each components’ CoM location, plus an additional factor which is derived in Appendix A. The resulting parameters for an 8 year-old child wearing the appropriately configured pediatric exoskeleton are shown in Table 6.

### 4.2. Equations of Motion

To apply HZD control, it is necessary to have a hybrid model of the patient-exoskeleton system. This hybrid model entails two parts: a continuous swing phase model representing the bulk motion of the system, and a discrete double support phase model representing an instantaneous impact event.

To derive the continuous swing phase model, Lagrangian mechanics is utilized assuming one foot is pinned to the ground. The Lagrangian ℒ can be calculated from the difference between kinetic energy T and potential energy U.

(8) ℒ=T−U

The potential energy can be calculated from the sum of the constituent parts $U_{i}=m_{i}gh_{i}$ where $m_{i}$ is the mass and $h_{i}$ is the height of the CoM of link i relative to the stance foot position. The height of the CoM of each link can be computed using forward kinematics in terms of the joint angles q. The kinetic energy is composed of not only the translational motion but also rotational motion of the links. The translational energy can be calculated as the sum of the constituent parts $T_{i}^{T}=m_{i}v_{i}^{2}/2$ where $v_{i}$ is the speed of the CoM of link i, which is similarly calculated using forward kinematics. The rotational energy can be calculated again as the sum of the constituent parts $T_{i}^{R}=I_{i}\omega_{i}^{2}/2$ where $I_{i}$ is the moment of inertia about the CoM of link i. Since the system is assumed to be planar, the angular velocity $\omega_{i}$ of link i can be calculated as simply the sum of the appropriately chosen elements of the joint angular velocity vector $\dot{q}$ with signs introduced based on sign convention.

With the Lagrangian found in terms of q and $\dot{q},$ the equations of motion for link i=1…5 for the swing phase can be derived through the application of the Euler-Lagrange equations of the second kind

(9) $$\frac{d}{dt}\left(\frac{\partial L}{\partial {\dot{q}}_{i}}\right)-\frac{\partial L}{\partial q_{i}}=w_{i}$$

where $w_{i}$ is the generalized inputs to the system acting on joint *i*. The resultant equations of motion can be expressed in standard form for robotic systems

(10) $$M\left(q\right)\ddot{q}+C\left(q,\dot{q}\right)\dot{q}+G\left(q\right)=Bu$$

where q is the joint coordinate vector, M(q) is the inertia matrix, $C\left(q,\dot{q}\right)$ is the Coriolis-centrifugal matrix, G(q) is the vector of gravitational torques, and u is the vector of input torques which are related to the generalized inputs via w=Bu for the input remapping matrix B.

To derive the discrete stance phase model, the equations of motion for a floating body model are required. Following the same procedure as above, the floating body model is

(11) $$M_{e}\left(q_{e}\right){\ddot{q}}_{e}+C_{e}\left(q_{e},{\dot{q}}_{e}\right){\dot{q}}_{e}+G_{e}\left(q_{e}\right)=B_{e}u$$

where $q_{e}$ is the extended joint configuration vector defined as the hip coordinates concatenated with the joint angles q, $M_{e}\left(q_{e}\right)$ is the extended inertia matrix, $C_{e}\left(q_{e},{\dot{q}}_{e}\right)$ is the extended Coriolis-centrifugal matrix, $G_{e}\left(q_{e}\right)$ is the extended gravitational torque-force vector, and $B_{e}$ is the input remapping matrix for the extended model with rank equal to that of B.

Stance phase is assumed to be an instantaneous event occurring when the swing leg impacts the ground. Stance phase is modeled using discrete dynamics with a mapping from the pre-impact states $x^{-}=\left(q^{-},{\dot{q}}^{-}\right)$ to the post-impact states $x^{+}=\left(q^{+},{\dot{q}}^{+}\right)$ [33]. The joint angles are assumed to remain the same, and therefore, the configuration of the system is related through $q^{+}=\Delta_{q}q^{-}$ for a constant position remapping matrix $\Delta_{q}$ which swaps the stance leg and swing leg. The joint velocities are derived by assuming angular momentum is conserved, as derived in [33]. In extended coordinates, the post-impact velocities are related to the pre-impact velocities using

(12) $$\left[\begin{array}{c} {\dot{q}}_{e}^{+}\\ F^{T}\\ F^{N} \end{array}\right]={\left[\begin{array}{cc} D_{e}\left(q_{e}^{-}\right) & -{\left(\frac{\partial E_{e}}{\partial q_{e}}\right)}^{T}\\ -\frac{\partial E_{e}}{\partial q_{e}} & 0_{2\times 2} \end{array}\right]}^{-1}\left[\begin{array}{c} D_{e}\left(q_{e}^{-}\right){\dot{q}}_{e}^{-}\\ 0_{2\times 1} \end{array}\right]$$

along with the tangential and normal ground reaction impulse forces $F^{T}$ and $F^{N}$, respectively, where $E_{e}\;$is the swing foot Cartesian position. Since the stance foot is assumed to be pinned in the model, there is an invertible relation between the extended model states and pinned model states. As such, the extended model states $q_{e}\;,\;{\dot{q}}_{e}$ can be calculated from the pinned model states $q,\;\dot{q}$. Subsequently, these can be used to find the extended model joint velocities post-impact ${\dot{q}}_{e}^{+}$ and in turn, pinned model joint velocities ${\dot{q}}^{+}$. Altogether, these computations can be combined as ${\dot{q}}^{+}=\Delta_{\dot{q}}\left(q^{-}\right){\dot{q}}^{-}$ where $\Delta_{\dot{q}}$ is the configuration-dependent velocity remapping matrix.

The hybrid model consists of continuous dynamics representing the swing phase and discrete dynamics representing the stance phase. Combining these together, the hybrid model is:

(13) $$\begin{array}{cc} \dot{x}=f\left(x\right)+g\left(x\right)u, & x^{-}\notin S\\ x^{+}=\Delta \left(x^{-}\right) & x^{-}\in S \end{array}$$

where Δ is the remapping function combining the effects from $\Delta_{q}\;$and$\;\Delta_{\dot{q}}$, and

(14) $$S=\left\{\left(q,\dot{q}\right):E_{h}\left(q\right)>0\wedge E_{v}\left(q\right)=0\right\}$$

is the impact manifold where $E_{h}$ and $E_{v}$ are the swing foot horizontal and vertical positions, respectively. The manifold S represents the states where the swing foot hits the ground and designates when the discrete dynamics occur.

## 5. Hybrid Zero Dynamics Control

A zero dynamics-based controller drives the system towards a manifold and renders the manifold invariant. In this paper, the manifold was defined as the set of functions

(15) $$e\left(q\right)=H_{0}q-H\left(s\left(q\right)\right)$$

being equal to zero where $H_{0}$ remaps the configuration variables to their corresponding constraint functions h and the constraint functions yield desired robot configuration values based on the normalized phase variable s. In HZD, s is a strictly monotonically increasing scalar representing the progression of gait and is normalized to be from 0 to 1. When the system states are on the manifold so that e(q)=0, the system is said to satisfy the virtual constraints and the dynamics are of order lower than that of the system.

For this paper, the virtual constraints were defined using the joint angles corresponding with the actuators, and therefore, $H_{0}=B$. The normalized phase was defined as

(16) $$s=\frac{\theta -\underline{\theta }}{\bar{\theta }-\underline{\theta }}$$

where non-normalized scalar phase variable $\theta =cq_{e}\in \left[\underline{\theta },\bar{\theta }\right]\;$is the vector product of the row vector *c* (1×7) and the extended joint configuration column vector $q_{e}$ (7×1). The vector *c* was chosen such that its vector product with the extended joint configuration vector is equal to the horizontal coordinate of the hip in the anterior-posterior direction relative to the location of the stance foot. The location of the stance ankle was taken to be the origin and zero point, as shown in Figure 4. The trajectory optimization in CasADi (TROPIC) toolbox for MATLAB was utilized for running a direct collocation optimization to solve for a set dynamically stable joint profiles to serve as a phase-based trajectory reference [34]. The bounds $\bar{\theta },\underline{\theta }$ were defined as the maximum and minimum value of θ at the end and beginning of the generated gait cycle, respectively, so that the normalized phase *s* sweeps from 0 to 1 throughout locomotion. Here, h was defined as a set of interpolation tables mapping the normalized phase variable to the TROPIC-generated trajectories.

Typically, cost of transport or torque is minimized in the optimization. Although gaits resulting from the optimization using such an objective function are dynamically stable, they do not necessarily resemble nominal gait patterns observed in healthy ambulation. Such a gait profile may not be ideal for application in therapeutic intervention of individuals with gait disabilities. Instead, similar to [35], this paper applied HZD for gait guidance by referencing human gait and choosing an objective function as

(17) $$J=t_{f}\underset{i}{\sum }e_{i}^{T}\sqrt{W}e$$

where $e_{i}$ is the error between the optimized trajectory and human reference trajectory for *i* in a range of collocation indices for the stance hip, stance knee, swing hip, and swing knee. This objective function allows for the zero dynamics manifold resulting from TROPIC to exhibit gait patterns similar to that of healthy individuals. The weight matrix *W* is a positive definite diagonal matrix with elements manually chosen for yielding adequate performance across the joints. Optimization constraints were included to limit stride length and walking speed within two standard deviations of the mean as reported in Table 5.

The TROPIC toolbox generates an optimal phase-based reference trajectory, which is then applied to the simulated human-exoskeleton system through the application of a phase-based PD control law. In prior simulation work, a proportional-derivative (PD) control law sufficed for achieving adequate performance [36]. Additionally, it was shown that high-gain PD controllers can yield locally stable periodic gaits in HZD controllers [37]. This paper applied a phase-based PD control strategy for approaching the manifold

(18) $$u=Pe+D\dot{e}$$

where P and D are positive definite diagonal gain matrices manually tuned for achieving adequate performance. The errors e and its derivative with respect to time $\dot{e}$ were taken to be the difference between the robot’s configuration and the desired configuration as calculated by the phase-based trajectory reference calculated in simulation.

It is important to note that the simulated system does not consider the effect of non-instantaneous double-support phases. While the hybrid model in both HZD control and in this simulation assumes an instantaneous double-support phase, this is not reflective of real human gait which exhibits non-instantaneous double-support phases. In the authors’ previous work, HZD control methods were employed on an adult human-exoskeleton system by adjusting the controller implementation to allow for easy transitions across the double-support phases [23,24]. However, the adjustment to control made in those publications were unnecessary for this simulation, as the controller and simulation utilizes an instantaneous double-support phase model.

## 6. Simulation Results

**Figure 6:**
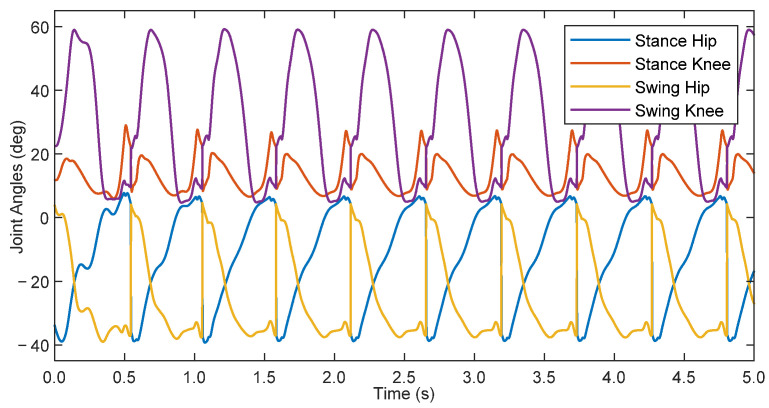
Joint angles of an 8 year-old child walking in simulation with the pediatric exoskeleton.

With the model equations and parameters identified for the patient-exoskeleton system, and with the information about nominal healthy gait of children as provided by Schwartz et al. [15], the TROPIC toolbox was used to derive a target zero dynamics manifold for the average 8 year-old child using the pediatric exoskeleton. The resultant manifold yielded reference joint positions in terms of a state-dependent phase variable, which were then used as targets for the phase-based PD control law defined in Equation (18). The proportional gains for the stance hip, stance knee, swing hip, and swing knee were 9.0, 6.0, 4.5, and 3.0 kNm/rad, respectively, whereas the derivative gains were 15 Nm·s/rad across all joints. A dynamic simulation of this system was initialized on the zero dynamics manifold and run for 5 s, and the results are shown in Figure 6.

The simulation initially had transients which decayed over the first 3 steps, after which the system settled on a gait cycle with a step period of 0.538 s. This is slightly longer than the optimized zero dynamic manifold at 0.518 s. This difference arose as a result of errors in tracking performance, which can affect the periodicity of the system. Additionally, there was a discrepancy between the input-output feedback linearization controller used in the TROPIC optimization during the generation of the ideal gait profile vs. the phase-based PD controller utilized in this simulation. The difference in applied controllers helps explain the existence of errors in the simulation despite initializing the simulation along the zero dynamics manifold. The root-mean-square (RMS) errors, with respect to the phase-based calculated reference, after reaching steady state were 1.42, 1.07, 1.10, and 0.77 deg for the stance hip, stance knee, swing hip, and swing knee, respectively. With respect to the full scale of joint angle ranges for each joint, this represents error percentages of 3.26% 3.74%, 2.68%, and 1.20% for the stance knee, swing hip, and swing knee, respectively. Although these are small, they can contribute to the difference in step period. Ultimately, the PD controller was capable of adequately following the manifold to achieve a dynamically stable walking gait. 

**Figure 7:**
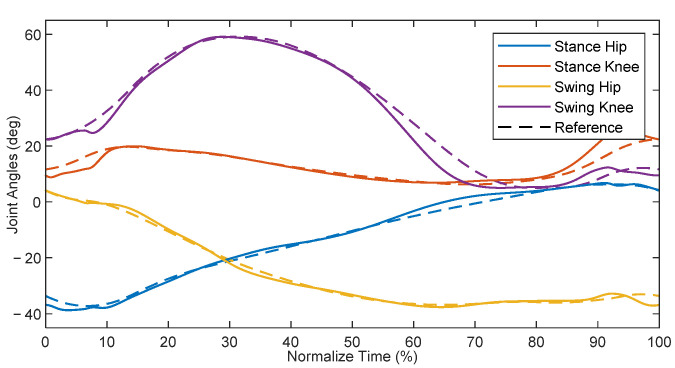
Steady-state gait cycle compared to time-based TROPIC-optimized trajectory.

A comparison of the steady-state joint angles from the simulation to the ideal TROPIC-optimized trajectory can be found in Figure 7, plotted with respect to normalized time. The resulting gait pattern from simulation qualitatively resembled that of the desired trajectory. Quantifying the differences between the two, the RMS errors were 1.30, 2.35, 1.05, and 2.95 deg for the stance hip, stance knee, swing hip, and swing hip, respectively. Expressed as a percentage of the full-scale range of joint angles, this represents error percentages of 2.99% 8.21%, 2.56%, and 4.59% for the stance knee, swing hip, and swing knee, respectively. The discrepancy between the errors with respect to the in-simulation phase-based calculated reference and the errors with respect to the TROPIC-optimized trajectory suggests that the evolution of the phase variable, which in turn defines the in-simulation calculated reference, was evolving slightly differently than what was expected in the TROPIC optimization. However, the periodic behavior shown in Figure 6 suggests that these discrepancies did not prevent a dynamically stable walking pattern from emerging. These results are promising, as they suggest that a HZD-based controller can provide gait guidance and thus may be useful for pediatric rehabilitation.

## 7. Conclusions

This paper presented the latest design iteration of an adjustable pediatric exoskeleton, and applied a HZD-based controller onto said system with an 8 year-old model patient in simulation. The pediatric exoskeleton was designed to actuate the hip and knee joints, and was able to accommodate the expected range of anthropometrics for children 6 to 11 years old. The adjustment mechanisms require minimal disassembly for refitting, and the exoskeleton should weigh roughly 3.5 kg excluding internal electronics, miscellaneous wires, and other minor components. The HZD optimization and resultant zero dynamics manifold were designed to provide gait guidance by emulating nominal healthy gait patterns of children. The phase-based approach for providing gait guidance lends itself for eventual application in exoskeleton-assisted gait rehabilitation. The results showed that the controller, with no input from the subject, was able to achieve dynamically stable walking gaits while following a human-like gait pattern. These preliminary results suggest that the HZD controller is a promising control candidate for gait guidance and eventual experimental application for rehabilitation in children with gait abnormalities.

Moving forward, the design of the adjustable pediatric exoskeleton must be finalized, fabricated, and validated, initially in healthy children within the target age range. Once the pediatric exoskeleton design has been proven to be compatible with the target age range in simple walking experiments, the authors aim to conduct a multi-subject clinical study using a HZD-based controller for children with gait abnormalities. Additionally, the pediatric exoskeleton designed and described in this manuscript can serve as a testbed for other rehabilitative exoskeleton controllers for pediatric subjects, including controllers which consider factors beyond gait stability such as for operator safety and device torque magnitude reduction [38]. 

## Figures and Tables

**Table 1:** Patient gross anthropometrics (mean ± standard deviation).

Age (yr)	Weight (kg)	Height (cm)
6	23.9 ± 5.2	118.8 ± 6.2
8	31.6 ± 9.3	132.1 ± 8.7
11	46.5 ± 20.5	149.3 ± 11.2

**Table 2:** Patient body segment parameters for an average 8 year-old child.

	HAT	Thigh	Shank
Length (m)	0.621	0.324	0.377
Mass (kg)	21.42	3.16	1.93
CoM (m)	0.389	0.140	0.228
Inertia (kg·m^2^)	2.0318	0.0345	0.0473

CoM is relative to hip joint for HAT and thigh, and is relative to knee joint for shank.

**Table 3:** Exoskeleton adjustment and anthropometric ranges.

	Hip Width (Min-Max cm)	Thigh Length (Min-Max cm)	Shank Length (Min cm)
Anthropometric Range	20.3–25.8	29.4–36.8	29.5
Adjustability Range	20.7–27.0	27.8–41.8	17.2

**Table 4:** Exoskeleton component properties.

	Hip Cradle All Ages	Thigh 6 Years Old	Thigh 8 Years Old	Thigh 11 Years Old	Shank All Ages
Mass (kg)	0.775	1.244	1.244	1.244	0.138
CoM (m)	0.097	0.150	0.167	0.188	0.115
Inertia (kg·m^2^)	0.0020	0.0127	0.0159	0.0208	0.0003

CoM is relative to hip joint for HAT and thigh, and is relative to knee joint for shank.

**Table 5:** Gait parameters (mean ± standard deviation).

Age (yr)	Speed (m/s)	Step Length (m)	Step Period (s)
6	1.067 ± 0.064	0.478 ± 0.028	0.449 ± 0.030
8	1.125 ± 0.068	0.532 ± 0.031	0.473 ± 0.031
11	1.196 ± 0.072	0.601 ± 0.035	0.503 ± 0.033

**Table 6:** Link properties of the human-exoskeleton system for an 8 year-old child.

	Upper Body	Thigh	Shank
Length (m)	0.621	0.324	0.377
Mass (kg)	22.20	4.60	2.07
CoM (m)	0.379	0.148	0.221
Inertia (kg·m^2^)	2.0973	0.0511	0.0492

Upper body is the patient HAT with exoskeleton hip cradle. Shank includes patient foot.

**Figure A1:**
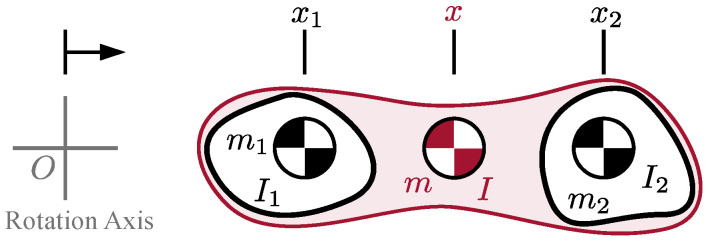
Combining inertias of two rigid bodies.

## Data Availability

The data presented in this study are available on request from the corresponding author.

## Appendix A

Consider the planar system in Figure A1, which is a connection between two rigid bodies, each with some moment of inertia about their respective CoM. With the two bodies rigidly connected, the combined moment of inertia *I* about the system CoM *x* is greater than the sum of the intrinsic inertia of the components. This is calculated in Equation (7) and is derived below.

The moment of inertia $I^{O}$ of the entire system about an arbitrary rotation axis O should be independent of whether it is calculated as two separate bodies or one single body. Using the parallel axis theorem, the calculation as a single combined body is

(A1) $$I^{O}=I+mx^{2}$$

where the m and x is the mass and CoM of the entire system relative to O, respectively. The calculation as two separate bodies is

(A2) $$I^{O}=I_{1}^{O}+I_{2}^{O}=I_{1}+m_{1}x_{1}^{2}+I_{2}+m_{2}x_{2}^{2}$$

By equating these two expressions for the inertia and substituting in the calculation for mass in Equation (5) and CoM in Equation (6), it is possible to derive the formula for the moment of inertia as Equation (7).

(A3) $$\begin{array}{l} I+mx^{2}=I_{1}+m_{1}x_{1}^{2}+I_{2}+m_{2}x_{2}^{2}\\ I+\left(m_{1}+m_{2}\right){\left(\frac{m_{1}x_{1}+m_{2}x_{2}}{m_{1}+m_{2}}\right)}^{2}=I+m_{1}x_{1}^{2}+I_{2}+m_{2}x_{2}^{2}\\ I+\frac{m_{1}^{2}x_{1}^{2}+2m_{1}m_{2}x_{1}x_{2}+m_{2}^{2}x_{2}^{2}}{m_{1}+m_{2}}=I+m_{1}x_{1}^{2}+I_{2}+m_{2}x_{2}^{2} \end{array}$$

From here, the equation can be rearranged to solve for the moment of inertia and then simplified.

(A4) $$\begin{array}{c} I=\frac{\left(m_{1}+m_{2}\right)\left(I_{1}+I_{2}\right)m_{1}^{2}x_{1}^{2}+m_{1}m_{2}x_{1}^{2}+m_{1}m_{2}x_{2}^{2}+m_{2}^{2}x_{2}^{2}}{m_{1}+m_{2}}\\ -\frac{m_{1}^{2}x_{1}^{2}+2m_{1}m_{2}x_{1}x_{2}+m_{2}^{2}x_{2}^{2}}{m_{1}+m_{2}}\\ \;\;I=\frac{\left(m_{1}+m_{2}\right)\left(I_{1}+I_{2}\right)+m_{1}m_{2}{\left(x_{1}-x_{2}\right)}^{2}}{m_{1}+m_{2}}\\ \;\;\;\;\;I=I_{1}+I_{2}+\frac{m_{1}m_{2}}{m_{1}+m_{2}}{\left(x_{1}-x_{2}\right)}^{2} \end{array}$$

This concludes the derivation of the formula for Equation (7).
